# Uncovering the Key Influencers of Healthy Lifestyle Behaviors in Patients With Epilepsy: A Cross‐Sectional Study

**DOI:** 10.1002/brb3.71371

**Published:** 2026-03-31

**Authors:** Nuray Bingöl, Bahar Çiftçi

**Affiliations:** ^1^ Department of Internal Medicine Nursing Ataturk University Erzurum Turkey; ^2^ Department of Fundamentals of Nursing Ataturk University Erzurum Turkey

**Keywords:** epilepsy, health‐promoting lifestyle, HPLP‐II, nursing, physical activity

## Abstract

**Background:**

Epilepsy is a chronic neurological disorder that affects daily functioning beyond seizure control. Adopting health‐promoting lifestyle behaviors is essential for effective self‐management; however, evidence on the key modifiable factors influencing these behaviors in adults with epilepsy remains limited. This study aimed to identify determinants of health‐promoting lifestyle behaviors and inform nurse‐led lifestyle interventions.

**Methods:**

This cross‐sectional descriptive study included 160 adults with epilepsy attending a university hospital in eastern Turkey (November 2023–May 2024). Data were collected using a sociodemographic/clinical questionnaire and the health‐promoting lifestyle profile II (HPLP‐II), which assesses health responsibility, physical activity, nutrition, spiritual growth, interpersonal relations, and stress management. Group comparisons were performed using *t*‐tests and one‐way ANOVA, and independent predictors of HPLP‐II total score were examined using multiple linear regression.

**Results:**

The mean HPLP‐II total score was 124.07 ± 25.49, indicating an overall moderate level of health‐promoting lifestyle behaviors. Subscale scores were highest for interpersonal relations (25.04 ± 5.59) and spiritual growth (24.72 ± 5.98), whereas physical activity was the lowest domain (13.74 ± 4.99). Nutrition scores (19.99 ± 4.97) were also lower than those for interpersonal relations and spiritual growth. Higher total scores were observed among women, married participants, those with higher education, and those with better perceived health status and diet adherence (*p* < 0.05). In the regression model, gender, self‐rated health status, diet adherence, and perceived diet status significantly predicted HPLP‐II total score (*R* ^2^ = 0.360; *F* = 3.501, *p* = 0.001).

**Conclusion:**

Adults with epilepsy demonstrated only moderate engagement in health‐promoting behaviors, with physical activity emerging as the most vulnerable domain. Targeted, nurse‐led interventions that include structured lifestyle education, individualized physical activity planning, diet adherence monitoring, and medication adherence counseling may help overcome condition‐specific barriers and improve long‐term self‐management.

## Graphical abstract

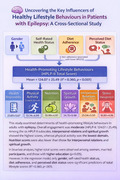

## 1 Introduction

Epilepsy is a chronic neurological condition characterized by recurrent seizures caused by abnormal electrical activity in the brain (Liu et al. 2023). Affecting approximately 70 million people globally, with a prevalence of up to 1%, epilepsy is a significant public health concern (Altan 2024; Eslamian et al. 2024). Beyond seizures, individuals face psychosocial and physical challenges, including injury risks, social stigma, and restricted participation in daily and professional life, which severely impact their quality of life (Fisher et al. 2014). While medical treatment is crucial, comprehensive lifestyle modifications are equally essential for improving overall well‐being (Szałwińska et al. 2021; Sauls et al. 2024; Yel and Karadakovan 2021; Rabiei et al. 2022). These behaviors encompass key dimensions, including nutrition, physical activity, health responsibility, stress management, interpersonal relations, and spiritual growth (Walker et al. 1987; Esin 2015). Such habits are integral to controlling seizures, building resilience against the challenges of epilepsy, and fostering holistic well‐being (Du Rausas et al. 2024).

Nutrition plays a vital role in the health of individuals with epilepsy. Studies reveal frequent deficiencies in key micronutrients, including vitamin B, D, zinc, and selenium, which are essential for neurological and general health (Szałwińska et al. 2021; Al Mheid and Quyyumi 2017). For instance, vitamin D deficiency, exacerbated by antiseizure medication use, increases the risk of osteoporosis (Theochari and Cock 2019). Moreover, epilepsy patients often face diet‐related issues such as dyslipidemia and cardiovascular diseases (Du Rausas et al. 2024). The ketogenic diet, a high‐fat, low‐carbohydrate regimen, has been shown to reduce seizure frequency, particularly in drug‐resistant cases (Liu et al. 2018; D'Andrea Meira et al. 2019). While effective, its long‐term impact on quality of life warrants careful consideration (Al Mheid and Quyyumi 2017). Addressing dietary habits and correcting micronutrient deficiencies are therefore critical components of epilepsy management (Du Rausas et al. 2024). Micronutrients such as vitamin D, B‐complex vitamins (particularly B6 and folate), magnesium, zinc, and selenium play critical roles in neuronal membrane stability, neurotransmitter synthesis, and the regulation of oxidative stress (Szałwińska et al. 2021; Al Mheid and Quyyumi 2017). Deficiencies in these nutrients may alter gamma‐aminobutyric acid (GABA) metabolism, increase neuronal excitability, and lower seizure threshold. Moreover, imbalances in glucose metabolism and insulin regulation may influence cortical excitability (Liu et al. 2018; D'Andrea Meira et al. 2019; Sarlo et al. 2023). Therefore, macro‐ and micronutrient balance may directly affect seizure frequency and neurological stability, ultimately influencing quality of life in individuals with epilepsy (Szałwińska et al. 2021; Du Rausas et al. 2024; Al Mheid and Quyyumi 2017).

Physical activity plays a crucial role in enhancing the quality of life and seizure control in individuals with epilepsy (Pimentel et al. 2015). However, fear of seizures often leads to sedentary lifestyles (Sauls et al. 2024; Talo and Turan 2023). Research indicates that regular, moderate physical activity—such as walking, yoga, and relaxation exercises—can reduce seizure frequency, decrease stress, and enhance general health (Talo and Turan 2023; Collard and Ellis‐Hill 2017). Talo and Turan's (2023) study highlighted the benefits of progressive muscle relaxation exercises in alleviating depressive symptoms in patients with epilepsy. To ensure safety, exercise plans should be tailored to seizure type and severity, avoiding overly strenuous activities (Pimentel et al. 2015; Collard and Ellis‐Hill 2017). The role of physical activity in reducing seizure frequency and boosting self‐confidence underscores its indispensability in epilepsy management (Pimentel et al. 2015; Talo and Turan 2023). Regular moderate physical activity may influence seizure control through several physiological mechanisms, including improved cerebral blood flow, modulation of hypothalamic–pituitary–adrenal (HPA) axis activity, reduction of circulating stress hormones such as cortisol, enhanced endorphin release, and improved autonomic balance. Exercise has also been associated with improved mitochondrial function, reduced neuroinflammation, and better regulation of excitatory–inhibitory neurotransmitter balance (Pimentel et al. 2015; Talo and Turan 2023; Collard and Ellis‐Hill 2017). By improving sleep quality and psychological resilience, physical activity may indirectly reduce seizure triggers and enhance overall neurological stability.

Health responsibility involves individuals actively protecting and improving their health (Dayapoğlu et al. 2021). Enhancing health responsibility in epilepsy patients is crucial for seizure control and better disease management (Sauls et al. 2024; Yel and Karadakovan 2021; Dayapoğlu et al. 2021). Spiritual growth and stress management are important psychosocial dimensions in epilepsy care (Du Rausas et al. 2024). Stress is a significant trigger for seizures in epilepsy patients, adversely affecting disease progression (Özcan and Çiftçi 2024). Learning and applying stress‐management techniques, such as meditation, breathing exercises, and yoga, can effectively reduce seizure frequency (Sauls et al. 2024). Strengthening social support systems and interpersonal relations further enhances patients’ quality of life (Tsigebrhan et al. 2021). Strengthening these relationships enhances psychological well‐being and encourages active participation in society (Yogarajah and Mula 2019).

Given the multidimensional nature of lifestyle behaviors in epilepsy, nurses play a key role in the adoption of healthy lifestyle behaviors by patients with epilepsy. Nurses guide patients on topics such as nutrition, physical activity, stress management, and substance use, and enhance individuals' health responsibilities by providing education on these areas (Yel and Karadakovan 2021; Dayapoğlu et al. 2021; Özcan and Çiftçi 2024). Nurse education and support for patients have a critical impact on epilepsy management. The originality of this study lies in its contribution to the nursing literature, as it examines the healthy lifestyle behaviors of individuals with epilepsy. Therefore, this study aimed to comprehensively evaluate health‐promoting lifestyle behaviors in adults with epilepsy and to identify key demographic and clinical predictors influencing these behaviors, thereby providing evidence to guide structured, nurse‐led lifestyle interventions.

**Research Questions**:

1. What is the level of healthy lifestyle behaviors of epilepsy patients?
2. How do the demographic characteristics of epilepsy patients affect their healthy lifestyle behaviors?

## 2 Methods

**Type of Research**: This study employed a cross‐sectional descriptive design.

**Place and Time of the Study**: Data were collected in a university hospital in eastern Turkey between November 2023 and May 2024.

**Population and Sample of the Study**: The study population consisted of patients aged 18 years and older with epilepsy who had attended the Neurology Outpatient Clinic of a university hospital in eastern Turkey for follow‐up within the previous year. Patients were required to have received their epilepsy diagnosis at least 1 year prior to participation. Patients were not specifically classified according to epilepsy subtype or treatment response status, and no separate sampling was performed for refractory epilepsy. The study aimed to evaluate health‐promoting lifestyle behaviors in adults with a general diagnosis of epilepsy attending routine outpatient follow‐up rather than focusing on specific clinical subtypes.

**Inclusion criteria** were: (1) age ≥18 years, (2) confirmed epilepsy diagnosis for at least 1 year, (3) ability to communicate in Turkish, (4) literacy, and (5) willingness to participate voluntarily.

**Exclusion criteria** were: (1) presence of severe cognitive impairment preventing questionnaire completion, (2) acute medical instability during the clinic visit, and (3) refusal to participate.

According to hospital records, 178 patients met the eligibility criteria. No sampling method was applied, and an attempt was made to reach the entire accessible population (census sampling). During the data collection period, 160 patients agreed to participate and completed the questionnaires, yielding a participation rate of 89.8%.

**Data Collection Tools**: “Questionnaire Form” and “Health‐Promoting Lifestyle Profile II (HPLP‐II)” were used to collect the data.

**Questionnaire Form**: The questionnaire, developed in accordance with the literature, consisted of 22 items. It included sociodemographic variables (age, gender, marital status, education level, and income status) and clinical variables (seizure frequency, disease duration, antiseizure medication use, and seizure control status). Body mass index (BMI) was calculated using the standard formula (kg/m^2^), with body weight and height reported by the participant during the interview.

**Health‐Promoting Lifestyle Profile II (HPLP‐II)**: The HPLP‐II was developed by Walker et al. in 1987 and revised in 1996 (Esin 2015). The Turkish validity and reliability study was conducted by Du Rausas et al. (2024), and permission to use the Turkish version was obtained from the authors. The scale consists of 52 positively worded items across six subscales: health responsibility, physical activity, nutrition, spiritual growth, interpersonal relations, and stress management. Items are rated on a 4‐point Likert scale from 1 (never) to 4 (regularly). Total scores range from 52 to 208, with higher scores indicating more frequent health‐promoting lifestyle behaviors. The original scale has a Cronbach's alpha of 0.94, and subscale alphas range from 0.79 to 0.87 (Du Rausas et al. 2024). In the present study, internal consistency reliability was high, with a Cronbach's alpha coefficient of 0.93 for the total scale. Subscale alpha values ranged from 0.66 to 0.81, indicating acceptable to good reliability.

**Data Collection**: The research data were collected through face‐to‐face interviews. Questionnaires were completed for the study participants, and each interview lasted approximately 10–15 min. Epilepsy patients were informed in detail about the research's aims and objectives, as well as the benefits to be gained from the study, and they provided written informed consent. Before the interview, patients with epilepsy were informed about the questionnaire's content and the interview process, and clear time‐management instructions were provided. Epilepsy patients participated in the study voluntarily, and their privacy and confidentiality were protected throughout the research process. All collected data were used only for research purposes, and personal information was kept confidential.

**Ethical Principles of the Study**: Approval was obtained from the Atatürk University Faculty of Medicine Ethics Committee before commencing the study (Board No: B.30.2.ATA.0.01.00/169; Date: April 15, 2021). The ethics approval remained valid during the data collection period. No protocol changes were made after the initial approval. Written consent was obtained after explaining the purpose, duration, and procedures to be performed during the study to patients who met the selection criteria. Patients enrolled in the study were informed that they were free to participate and could withdraw at any time. The patients from whom data would be collected were informed that their personal information would not be shared with others, and the “confidentiality principle” was observed.

### 2.1 Evaluation of the Data

Data analysis was performed using SPSS version 22.0 (IBM Corp., Armonk, NY, USA). Frequency and percentage analyses were used to evaluate descriptive characteristics, and mean scores and standard deviations were used to describe scale scores. Age was categorized into two groups (18–25 years and ≥26 years) to allow comparison between younger and older adults and to ensure adequate group sizes for statistical analysis based on the sample distribution. Normality was assessed using skewness and kurtosis values. Values between −1.5 and +1.5 were considered indicative of approximate normal distribution. Independent‐samples *t*‐test and one‐way ANOVA were used for group comparisons.

To identify independent predictors of health‐promoting lifestyle behaviors, multiple linear regression analysis was performed. The total HPLP‐II score was entered as the dependent variable. Independent variables included gender, marital status, education level, income status, seizure frequency, disease duration, self‐rated health status, diet adherence, perceived diet status, and antiseizure medication use. All independent variables were entered simultaneously into the regression model using the enter method, regardless of bivariate significance. Multicollinearity was assessed using variance inflation factor (VIF) values, and assumptions of linearity and homoscedasticity were evaluated prior to model interpretation. Internal consistency reliability was assessed using Cronbach's alpha. The statistical significance level was set at *p* < 0.05.

## 3 Results

Among the participants, 60.0% were aged ≥26 years, 71.3% were female, 42.5% had a university degree, and 73.1% lived in a provincial center (Table 1). Clinically, 59.4% had epilepsy for ≥6 years, 56.3% had an unidentified seizure type, and 75.6% reported not engaging in sports (Table 1).

**TABLE 1:** Distribution of descriptive characteristics of patients (*n* = 160).

Features	Variables	*N*	%
Age	18–25	64	40.0
	26 and above	96	60.0
Gender	Female	114	71.3
	Male	46	28.7
Marital status	Married	68	42.5
	Single	92	57.5
Education Status	Primary school	41	25.6
	High school	51	31.9
	University	68	42.5
Place of Settlement	Village	12	7.5
	District	31	19.4
	Province	117	73.1
Profession	Housewife	42	26.3
	Student	34	21.3
	Officer	31	19.4
	Retired worker	18	11.3
	Self‐employed	35	21.9
Income Status	Bad	65	40.6
	Moderate	90	56.3
	Good	5	3.1
Duration of epilepsy seizures (years)	1–5	65	40.6
	6 and above	95	59.4
Seizure frequency in the last 6 months	Once a week	42	26.3
	Once a month	48	30.0
	Once a year	70	43.8
Seizure type	Generalized tonic–clonic	51	31.9
	Partial seizure	19	11.9
	Unidentified seizure	90	56.3
Recognition of a seizure	Yes	89	55.6
	No	71	44.4
Number of medicines required to be used due to epilepsy	1	60	37.5
	2	57	35.6
	3	34	21.3
Self‐rated health status	Bad	13	8.1
	Moderate	93	58.1
	Good	54	33.8
Smoking status	Yes	49	30.6
	No	111	69.4
Alcohol use status	Yes	8	5.0
	No	152	95.0
Sports participation	Yes	39	24.4
	No	121	75.6
Adherence to diet status	Yes	56	35.0
	No	104	65.0
Sleep pattern	Bad	23	14.4
	Moderate	91	56.9
	Good	46	28.7
Perceived diet status	Bad	15	9.4
	Moderate	87	54.4
	Good	58	36.3
Weight	40–60kg	59	36.9
	61–75kg	55	34.4
	76 kg and over	46	28.7
Height	140–164 cm	81	50.6
	165 cm and above	79	49.4
BMI	Weak (below 18.5)	20	12.5
	Normal (between 18.5 and 24.9)	65	40.6
	Overweight (between 25 and 29.9)	55	34.4
	Obese (30 and over)	20	12.5

Health responsibility subscale score was 21.69 ± 5.67, physical activity was 13.74 ± 4.99, nutrition was 19.99 ± 4.97, spiritual growth was 24.72 ± 5.98, interpersonal relations was 25.04 ± 5.59, and stress management was 18.89 ± 4.86. The mean HPLP‐II total score was 124.07 ± 25.49 (Table 2). Taken together, the HPLP‐II total score reflects the combined contribution of all six subscales; therefore, lower scores in physical activity and nutrition likely pulled down the overall total despite relatively higher scores in interpersonal relations and spiritual growth. Health responsibility scores differed significantly according to age, gender, marital status, residence, income perception, diet adherence, perceived health, sleep pattern, and BMI (*p *< 0.05). These differences indicate that lifestyle engagement may not be uniformly distributed across demographic groups, highlighting potential inequality in self‐management capacity.

**TABLE 2:** Patients’ HPLP‐II total and subscale scores (mean ± SD) (*n* = 160).

	$\bar{X}$ ± SD	Min–max
Health responsibility	21.69 ± 5.67	9–36
Physical activity	13.74 ± 4.99	8–32
Nutrition	19.99 ± 4.97	9–33
Spiritual growth	24.72 ± 5.98	10–36
Interpersonal relations	25.04 ± 5.59	12–36
Stress management	18.89 ± 4.86	9–32
Dependent variable: HPLP‐II total score	124.07 ± 25.49	70–195

It was determined that the mean scores for “Physical Activity” were higher among single individuals, university graduates, and those who engaged in sports, and this difference was statistically significant (*p* < 0.05).

Nutrition scores differed significantly by age, marital status, education level, occupation, medication use, diet adherence, perceived diet status, weight, and BMI (*p* < 0.05).

It was determined that the mean scores of “Spiritual Growth” were higher in those whose income status perception was at a medium level, those who defined their health as good, those who described their sleep patterns as good, and those who described their diet as good, and this difference was statistically significant (*p* < 0.05).

Women, married individuals, those with a moderate income perception, those who described their health as good, those who described their sleep patterns as good, and those who described their diet as good had higher mean scores on interpersonal relations. This difference was statistically significant (*p* < 0.05).

Patients aged 26 years and older, university graduates, those who described their health as good, those who described their sleep patterns as good, and those who described their diet as good had higher mean scores in “Stress Management.” This difference was statistically significant (*p* < 0.05).

Patients aged 26 years and older, women, those with a university education level, married people, those with a medium level of income perception, those who defined their health as good, those who described their sleep patterns as good, and those who described their dietary patterns as good had higher mean scores on the “health‐promoting lifestyle profile II (HPLP‐II)”; high school graduates had lower mean scores, and this difference was statistically significant (*p* < 0.05) (Table 3).

**TABLE 3:** Comparison of the mean scores of the health‐promoting lifestyle profile‐II (HPLP‐II) and its subscales according to the descriptive characteristics of the patients (*n* = 160).

Features	Variables	Health responsibility	Physical activity	Nutrition	Spiritual growth	Interpersonal relations	Stress management	HPLP‐II total score
		$\bar{X}$ ± SD	$\bar{X}$ ± SD	$\bar{X}$ ± SD	$\bar{X}$ ± SD	$\bar{X}$ ± SD	$\bar{X}$ ± SD	$\bar{X}$ ± SD
**Age**	18–25	20.08 ± 5.06	14.28 ± 5.46	18.20 ± 3.99	23.73 ± 5.57	23.77 ± 4.88	17.97 ± 4.17	118.03 ± 22.37
	26 and above	22.76 ± 5.82	13.38 ± 4.66	21.18 ± 5.22	25.38 ± 6.18	25.90 ± 5.89	19.51 ± 5.19	128.09 ± 26.73
	**Test, *p***	***t* = ‐3.006** ***p* = 0.003**	*t* = 1.125 *p* = 0.262	***t* = ‐4.077** ***p* = 0.001**	*t* = ‐1.711 *p* = 0.089	***t* = ‐2.397** ***p* = 0.018**	***t* = ‐1.985** ***p* = 0.049**	***t* = ‐2.486** ***p* = 0.014**
**Gender**	Woman	22.48 ± 5.86	13.63 ± 4.78	20.30 ± 5.31	25.28 ± 5.99	25.81 ± 5.63	19.13 ± 4.87	126.63 ± 26.04
	Male	19.72 ± 4.66	14.00 ± 5.53	19.22 ± 3.97	23.33 ± 5.78	23.15 ± 5.07	18.30 ± 4.83	117.72 ± 23.13
	**Test, *p***	***t* = 2.855** ***p* = 0.005**	*t* = ‐0.421 *p* = 0.674	*t* = 1.408 *p* = 0.162	*t* = 1.887 *p* = 0.061	***t* = 2.776** ***p* = 0.006**	*t* = 0.975 *p* = 0.331	***t* = 2.022** ***p* = 0.045**
**Marital status**	Married	22.74 ± 5.98	12.51 ± 3.61	21.40 ± 5.55	25.75 ± 6.51	26.10 ± 6.36	19.35 ± 5.30	127.85 ± 27.20
	Single	20.91 ± 5.32	14.64 ± 5.66	18.95 ± 4.24	23.96 ± 5.46	24.26 ± 4.84	18.55 ± 4.50	121.27 ± 23.91
	**Test, *p***	***t* = 2.030** ***p* = 0.044**	***t* = ‐2.894** ***p* = 0.004**	***t* = 3.047** ***p* = 0.003**	*t* = 1.843 *p* = 0.068	***t* = 2.000** ***p* = 0.039**	*t* = 1.029 *p* = 0.305	*t* = 1.623 *p* = 0.107
**Education status**	Primary school^1^	22.17 ± 6.47	12.46 ± 4.77	22.12 ± 5.84	25.49 ± 7.19	25.95 ± 6.72	19.50 ± 4.56	128.00 ± 30.59
	High school^2^	20.76 ± 5.09	13.12 ± 3.95	18.43 ± 4.27	23.51 ± 5.42	23.53 ± 4.78	17.35 ± 3.83	116.71 ± 19.81
	University^3^	22.09 ± 5.56	14.97 ± 5.58	19.87 ± 4.47	25.16 ± 5.51	25.63 ± 5.25	19.80 ± 6.01	127.22 ± 25.09
	**Test, *p***	*F* = 0.995 *p* = 0.372	***F* = 3.939** ***p* = 0.021** **3‐1, 3‐2**	***F* = 6.756** ***p* = 0.002** **1–2**	*F* = 1.581 *p* = 0.209	*F* = 2.855 *p* = 0.061	***F* = 3.961** ***p* = 0.021** 3‐2, 2‐1	***F* = 3.223** ***p* = 0.042** 1–2, 2–3
**Place of settlement**	Village^1^	18.92 ± 5.30	10.42 ± 2.50	21.08 ± 5.73	24.83 ± 6.35	24.00 ± 7.46	17.25 ± 6.17	116.50 ± 28.83
	District^2^	20.10 ± 5.52	14.10 ± 5.45	18.16 ± 4.90	23.74 ± 5.14	24.26 ± 5.21	18.00 ± 4.96	118.35 ± 25.07
	Province^3^	22.39 ± 5.62	13.98 ± 4.97	20.36 ± 4.84	24.97 ± 6.16	25.36 ± 5.49	19.30 ± 4.66	126.36 ± 25.08
	**Test, *p***	***F* = 3.681** ***p* = 0.027** **3‐1, 3‐2**	*F* = 2.944 *p* = 0.056	*F* = 2.771 *p* = 0.066	*F* = 0.513 *p* = 0.600	*F* = 0.699 *p* = 0.499	*F* = 1.633 *p* = 0.199	*F* = 1.798 *p* = 0.169
**Profession**	Housewife^1^	23.57 ± 6.67	11.74 ± 3.04	22.21 ± 5.96	26.21 ± 6.69	26.74 ± 6.68	19.52 ± 5.66	130.00 ± 28.66
	Student^2^	20.35 ± 5.26	14.18 ± 5.84	17.97 ± 3.94	23.76 ± 5.29	24.74 ± 4.71	18.35 ± 3.89	119.35 ± 22.61
	Officer^3^	22.48 ± 5.93	15.94 ± 5.99	20.42 ± 5.07	25.10 ± 6.34	25.58 ± 5.61	19.94 ± 5.71	129.45 ± 28.95
	Retired worker^4^	19.50 ± 5.27	13.50 ± 4.74	20.17 ± 5.15	22.89 ± 6.61	22.78 ± 5.83	18.33 ± 5.31	117.17 ± 28.24
	Free^5^	21.14 ± 3.91	13.89 ± 4.46	18.80 ± 3.13	24.46 ± 4.79	24.00 ± 4.26	18.03 ± 3.37	120.31 ± 16.78
	**Test, *p***	***F* = 2.640** ***p* = 0.036** **1–2, 1–4**	***F* = 3.470** ***p* = 0.010** **1–2, 1–3**	***F* = 4.421** ***p* = 0.002** **1–2,1‐5, 2–3, 2–5**	*F* = 1.355 *p* = 0.252	*F* = 2.169 *p* = 0.075	*F* = 0.976 *p* = 0.422	*F* = 1.758 *p* = 0.140
**Income status**	Bad^1^	20.46 ± 6.00	13.45 ± 4.97	18.91 ± 4.34	22.52 ± 5.83	23.35 ± 5.65	17.86 ± 5.14	116.55 ± 25.89
	Moderate^2^	22.42 ± 5.33	13.73 ± 4.67	20.81 ± 5.30	26.20 ± 5.58	26.11 ± 5.24	19.59 ± 4.56	128.87 ± 23.90
	Good^3^	24.40 ± 4.67	17.60 ± 9.61	19.20 ± 4.60	26.60 ± 7.13	27.80 ± 6.14	19.80 ± 4.60	135.40 ± 28.29
	**Test, *p***	*F* = 2.198 *p* = 0.057	*F* = 1.618 *p* = 0.202	*F* = 2.900 *p* = 0.058	***F* = 8.053** ***p* = 0.001** **1–2**	***F* = 5.517** ***p* = 0.005** **1–2**	*F* = 2.525 *p* = 0.083	***F* = 5.172** ***p* = 0.007** **1–2**
**Duration of epileptic seizures**	1–5	21.71 ± 5.32	14.55 ± 5.91	19.95 ± 5.06	25.48 ± 5.87	25.37 ± 5.61	19.20 ± 4.89	126.26 ± 25.46
	6 and above	21.67 ± 5.92	13.18 ± 4.20	20.01 ± 4.93	24.20 ± 6.02	24.82 ± 5.59	18.68 ± 4.85	122.57 ± 25.53
	**Test, *p***	*t* = 0.037 *p* = 0.970	*t* = 1.616 *p* = 0.109	*t* = ‐0.071 *p* = 0.944	*t* = 1.330 *p* = 0.185	*t* = 0.608 *p* = 0.544	*t* = 0.659 *p* = 0.511	*t* = 0.900 *p* = 0.370
**Seizure frequency in the last 6 months**	Once a week	22.07 ± 5.37	14.62 ± 6.16	20.86 ± 4.70	24.76 ± 6.11	25.38 ± 5.98	19.19 ± 4.96	126.88 ± 26.45
	Once a month	20.83 ± 5.53	14.29 ± 5.34	19.42 ± 4.76	23.69 ± 4.89	24.04 ± 4.62	18.10 ± 5.28	120.38 ± 24.18
	Once a year	22.04 ± 5.95	12.83 ± 3.75	19.86 ± 5.26	25.40 ± 6.53	25.53 ± 5.93	19.26 ± 4.49	124.91 ± 25.85
	**Test, *p***	*F* = 0.777 *p* = 0.462	*F* = 2.139 *p* = 0.121	*F* = 0.983 *p* = 0.376	*F* = 1.173 *p* = 0.312	*F* = 1.113 *p* = 0.331	*F* = 0.908 *p* = 0.405	*F* = 0.796 *p* = 0.453
**Seizure type**	Generalized tonic–clonic	21.73 ± 5.59	13.94 ± 3.99	19.25 ± 4.34	24.55 ± 5.62	25.29 ± 5.20	18.37 ± 4.14	123.14 ± 23.62
	Partial seizure	20.37 ± 5.31	14.68 ± 5.35	20.05 ± 4.14	24.53 ± 4.93	24.42 ± 4.44	19.11 ± 4.77	123.16 ± 22.92
	Unidentified seizure	21.94 ± 5.80	13.42 ± 5.43	20.39 ± 5.44	24.86 ± 6.41	25.03 ± 6.04	19.14 ± 5.26	124.79 ± 27.20
	**Test, *p***	*F* = 0.605 *p* = 0.547	*F* = 0.560 *p* = 0.572	*F* = 0.847 *p* = 0.430	*F* = 0.053 *p* = 0.948	*F* = 0.167 *p* = 0.846	*F* = 0.429 *p* = 0.652	*F* = 0.081 *p* = 0.922
**Recognition of a seizure**	Yes	21.67 ± 5.68	13.66 ± 4.67	19.87 ± 4.83	24.58 ± 5.87	25.02 ± 5.46	18.98 ± 4.68	123.79 ± 24.14
	No.	21.70 ± 5.70	13.83 ± 5.41	20.14 ± 5.17	24.89 ± 6.14	25.07 ± 5.79	18.79 ± 5.10	124.42 ± 27.26
	**Test, *p***	*t* = ‐0.033 *p* = 0.974	*t* = ‐0.211 *p* = 0.833	*t* = ‐0.348 *p* = 0.729	*t* = ‐0.318 *p* = 0.751	*t* = ‐0.054 *p* = 0.957	*t* = 0.244 *p* = 0.808	*t* = ‐0.156 *p* = 0.876
**Number of medicines required to be used due to epilepsy**	1^2^	21.33 ± 6.28	13.28 ± 4.27	18.77 ± 4.39	25.30 ± 6.01	25.15 ± 5.39	18.97 ± 4.50	122.80 ± 24.46
	2^3^	22.46 ± 5.36	14.81 ± 6.05	19.97 ± 4.37	25.09 ± 6.06	25.68 ± 5.59	19.56 ± 5.15	129.16 ± 27.47
	3^4^	21.18 ± 5.55	12.74 ± 3.89	21.56 ± 5.60	22.85 ± 5.60	23.47 ± 5.96	17.50 ± 5.11	117.71 ± 25.51
	**Test, p**	*F* = 0.546 *p* = 0.652	*F* = 1.507 *p* = 0.215	***F* = 3.666** ***p* = 0.014** **2–4**	*F* = 1.436 *p* = 0.234	*F* = 1.295 *p* = 0.278	*F* = 1.345 *p* = 0.262	*F* = 1.529 *p* = 0.209
**Self‐rated health status**	Bad^1^	21.00 ± 4.86	12.62 ± 4.68	18.77 ± 4.34	20.38 ± 6.55	22.31 ± 6.60	15.54 ± 3.80	110.62 ± 23.48
	Moderate^2^	21.33 ± 5.47	13.22 ± 4.53	19.54 ± 4.90	23.81 ± 5.79	24.22 ± 5.34	18.29 ± 4.91	120.40 ± 24.57
	Good^3^	22.46 ± 6.18	14.91 ± 5.66	21.06 ± 5.13	27.33 ± 5.12	27.13 ± 5.18	20.74 ± 4.34	133.63 ± 24.79
	**Test, p**	*F* = 0.780 *p* = 0.460	*F* = 2.358 *p* = 0.133	*F* = 2.045 *p* = 0.133	***F* = 10.867** ***p* = 0.001** **1–3, 2–3**	***F* = 6.804** ***p* = 0.001** **1–3, 2–3**	***F* = 8.452** ***p* = 0.001** **1–3, 2–3**	***F* = 7.077** ***p* = 0.001** **1–3, 2–3**
**Smoking status**	Yes	20.94 ± 5.25	13.78 ± 4.96	19.39 ± 4.64	24.94 ± 5.81	24.55 ± 5.58	18.33 ± 4.55	121.92 ± 24.47
	No.	22.02 ± 5.83	13.72 ± 5.03	20.25 ± 5.11	24.62 ± 6.07	25.26 ± 5.60	19.14 ± 4.99	125.02 ± 25.98
	**Test, p**	*t* = ‐1.111 *p* = 0.268	*t* = 0.064 *p* = 0.949	*t* = ‐1.014 *p* = 0.312	*t* = 0.308 *p* = 0.758	*t* = ‐0.740 *p* = 0.460	*t* = ‐0.982 *p* = 0.328	*t* = ‐0.708 *p* = 0.480
**Alcohol use status**	Yes	23.63 ± 4.34	14.88 ± 7.22	20.38 ± 3.34	25.13 ± 5.00	25.88 ± 4.82	20.13 ± 3.94	130.00 ± 23.96
	No.	21.59 ± 5.72	13.68 ± 4.88	19.97 ± 5.05	24.70 ± 6.04	25.00 ± 5.64	18.83 ± 4.90	123.76 ± 25.60
	**Test, p**	*t* = 0.992 *p* = 0.323	*t* = 0.660 *p* = 0.510	*t* = 0.226 *p* = 0.822	*t* = 0.197 *p* = 0.844	*t* = 0.430 *p* = 0.667	*t* = 0.735 *p* = 0.464	*t* = 0.674 *p* = 0.501
**Doing sports**	Yes	21.90 ± 4.95	18.72 ± 5.42	20.18 ± 4.14	24.15 ± 5.43	24.13 ± 4.82	19.00 ± 4.45	128.08 ± 24.11
	No.	21.62 ± 5.90	12.13 ± 3.62	19.93 ± 5.23	24.90 ± 6.15	25.34 ± 5.80	18.86 ± 5.00	122.78 ± 25.88
	**Test, p**	*t* = 0.265 *p* = 0.791	***t* = 7.097** ***p* = 0.001**	*t* = 0.277 *p* = 0.782	*t* = ‐0.678 *p* = 0.499	*t* = ‐1.178 *p* = 0.241	*t* = 0.157 *p* = 0.876	*t* = 1.130 *p* = 0.260
**Diet adherence status**	Yes	23.41 ± 5.68	15.82 ± 5.80	21.27 ± 4.65	25.38 ± 5.84	25.59 ± 5.06	19.41 ± 4.76	130.88 ± 25.81
	No.	20.76 ± 5.47	12.62 ± 4.11	19.30 ± 5.02	24.37 ± 6.05	24.75 ± 5.86	18.62 ± 4.91	120.40 ± 24.67
	**Test, p**	***t* = 2.886** ***p* = 0.004**	***t* = 3.669** ***p* = 0.001**	***t* = 2.428** ***p* = 0.016**	*t* = 1.019 *p* = 0.310	*t* = 0.905 *p* = 0.367	*t* = 0.988 *p* = 0.325	***t* = 2.520** ***p* = 0.014**
**Describe your sleep pattern**	Bad^1^	20.78 ± 5.78	12.30 ± 3.13	18.35 ± 3.41	23.61 ± 5.72	23.87 ± 5.31	16.13 ± 3.88	115.04 ± 19.62
	Moderate^2^	21.21 ± 5.81	13.67 ± 4.80	19.00 ± 4.67	24.04 ± 6.03	24.45 ± 5.62	18.11 ± 4.51	120.48 ± 25.58
	Good^3^	23.09 ± 5.17	14.59 ± 5.96	22.76 ± 5.18	26.61 ± 5.68	26.80 ± 5.37	21.83 ± 4.63	135.67 ± 24.36
	**Test, p**	*F* = 2.046 *p* = 0.133	*F* = 1.633 *p* = 0.199	***F* = 11.565** ***p* = 0.001** **1–3, 2–3**	***F* = 3.374** ***p* = 0.037** **1–3, 2–3**	***F* = 3.402** ***p* = 0.036** **1–3, 2–3**	***F* = 15.767** ***p* = 0.001** **1–3, 2–3**	***F* = 7.711** ***p* = 0.001** **1–3, 2–3**
**Perceived diet status**	Bad^1^	20.13 ± 4.97	11.20 ± 2.76	17.13 ± 3.89	21.33 ± 4.62	22.73 ± 5.61	16.33 ± 4.73	108.87 ± 19.66
	Moderate^2^	20.79 ± 5.26	13.84 ± 4.77	19.03 ± 4.64	23.67 ± 5.75	23.97 ± 5.10	17.93 ± 4.22	119.23 ± 23.16
	Good^3^	23.43 ± 6.07	14.24 ± 5.61	22.16 ± 4.93	27.17 ± 5.80	27.26 ± 5.66	21.00 ± 5.07	135.26 ± 26.15
	**Test, p**	***F* = 4.589** ***p* = 0.012** 2–3	*F* = 2.285 *p* = 0.105	***F* = 10.766** ***p* = 0.001** **1–3, 2–3**	***F* = 9.573** ***p* = 0.001** **1–3, 2–3**	***F* = 8.123** ***p* = 0.001** **1–3, 2–3**	***F* = 10.338** ***p* = 0.001** **1–3, 2–3**	***F* = 11.070** ***p* = 0.001** **1–3, 2–3**
**Weight**	40–60kg	20.88 ± 5.70	13.61 ± 4.94	18.12 ± 3.87	23.83 ± 5.03	24.08 ± 4.61	17.93 ± 4.23	118.46 ± 21.11
	61‐75 kg	22.33 ± 5.88	13.89 ± 5.34	21.56 ± 5.39	25.25 ± 6.69	25.55 ± 6.37	19.58 ± 5.10	128.16 ± 28.38
	76 kg and over	21.96 ± 5.35	13.72 ± 4.74	20.50 ± 5.02	25.22 ± 6.18	25.67 ± 5.68	19.30 ± 5.20	126.37 ± 26.21
	**Test, p**	*F* = 0.999 *p* = 0.371	*F* = 0.045 *p* = 0.956	***F* = 7.794** ***p* = 0.001** **1–2, 1–3**	*F* = 1.033 *p* = 0.358	*F* = 1.389 *p* = 0.252	*F* = 1.894 *p* = 0.154	*F* = 2.367 *p* = 0.097
**Height**	140–164 cm	22.26 ± 5.93	13.38 ± 4.57	20.01 ± 4.88	24.75 ± 5.63	25.63 ± 5.42	18.89 ± 4.93	124.93 ± 24.16
	165 cm–above	21.10 ± 5.37	14.10 ± 5.40	19.96 ± 5.09	24.68 ± 6.35	24.44 ± 5.73	18.90 ± 4.81	123.19 ± 26.91
	**Test, p**	*t* = 1.295 *p* = 0.197	*t* = ‐0.909 *p* = 0.365	*t* = 0.064 *p* = 0.949	*t* = 0.073 *p* = 0.942	*t* = 1.346 *p* = 0.180	*t* = ‐0.013 *p* = 0.990	*t* = 0.430 *p* = 0.668
**BMI**	Weak^1^	19.95 ± 5.06	13.45 ± 4.44	17.85 ± 4.00	23.35 ± 5.53	24.10 ± 4.73	17.30 ± 4.12	116.00 ± 20.85
	Normal^2^	21.45 ± 5.82	14.65 ± 5.79	21.24 ± 5.04	24.49 ± 5.41	24.20 ± 5.28	18.92 ± 4.99	123.15 ± 25.78
	Overweight^3^	22.51 ± 5.85	13.15 ± 4.73	19.45 ± 4.98	25.73 ± 6.63	26.22 ± 6.02	19.44 ± 4.92	128.27 ± 26.62
	Obese^4^	21.95 ± 5.15	12.70 ± 2.70	20.45 ± 4.96	24.05 ± 6.27	25.50 ± 5.89	18.90 ± 4.93	123.55 ± 25.04
	**Test, p**	*F* = 1.067 *p* = 0.365	*F* = 1.291 *p* = 0.279	***F* = 2.797** ***p* = 0.042** **1–2, 3–2**	*F* = 0.986 *p* = 0.401	*F* = 1.554 *p* = 0.203	*F* = 0.947 *p* = 0.420	*F* = 1.202 *p* = 0.311

*Note*: The superscript numbers (1, 2, and 3) represent the ordering of the groups for comparison purposes. Differences between these groups are indicated in the table (e.g., 1–3, 2–3).

Table 4 shows that gender (*β* = −0.190, *p* = 0.041), self‐rated health status (*β* = 0.241, *p* = 0.004), diet adherence (*β* = −0.239, *p* = 0.003), and perceived diet status (*β* = 0.175, *p* = 0.030) were significant predictors of the HPLP‐II total score (*F* = 3.501, *p* = 0.001) (Table 4). These findings suggest that diet‐related perceptions and self‐rated health not only influence individual subdomains but also significantly shape the overall lifestyle behavior profile. Notably, the negative beta coefficient for diet non‐adherence (*β* = −0.239) indicates that lack of adherence substantially decreases overall lifestyle engagement.

**TABLE 4:** Regression analysis results regarding the effect of patients' descriptive characteristics on the level of healthy lifestyle behaviors (*n* = 160).

Dependent variable	Model	Variables	B	S. error	β	*t*	*p*
**Health‐Promoting Lifestyle Profile II**	1	Fixed	82.952	29.837		2.780	0.006
		Age	6.945	5.014	0.134	1.385	0.168
		Gender	−10.662	5.158	−0.190	−2.067	**0.041**
		Marital status	.790	5.041	0.015	0.157	0.876
		Education status	−1.745	2.515	−0.055	−0.694	0.489
		Place of settlement	4.132	2.976	0.100	1.388	0.167
		Profession	−1.236	1.450	−0.072	−0.852	0.396
		Income status	6.097	3.567	0.131	1.709	0.090
		Duration of epileptic seizures	−7.274	4.278	−0.141	−1.700	0.091
		Seizure frequency in the last 6 months	0.622	2.380	0.020	0.262	0.794
		Seizure type	1.550	2.136	0.055	0.726	0.469
		Recognition of a seizure	−1.277	3.701	−0.025	−0.345	0.731
		Number of medicines required to be used due to epilepsy	−2.122	2.284	−0.072	−0.929	0.355
		Self‐rated health status	10.289	3.481	0.241	2.956	**0.004**
		Smoking Status	3.746	4.282	0.068	0.875	0.383
		Alcohol use status	−0.141	8.445	−0.001	−0.017	0.987
		Sports participation	−2.788	4.595	−0.047	−0.607	0.545
		Adherence to diet status	−12.745	4.285	−0.239	−2.974	**0.003**
		Sleep pattern	4.750	3.277	0.120	1.449	0.150
		Perceived diet status	7.184	3.266	0.175	2.200	**0.030**
		Weight	5.260	4.332	0.167	1.214	0.227
		Height	1.998	4.474	0.039	0.447	0.656
		BMI	−0.725	3.939	−0.025	−0.184	0.854
	*R* = 0.600, *R* ^2^ = 0.360 *F* = 3.501, *p* = 0.001, DW = 2.152						

*Note*: Coding: Gender (0 = female, 1 = male); adherence to diet (0 = yes, 1 = no); self‐rated health status (1 = poor, 2 = moderate, 3 = good); perceived diet status (1 = poor, 2 = moderate, 3 = good).

## 4 Discussion

In this study, the healthy lifestyle behaviors of epilepsy patients were found to be at a moderate level (Table 2). This overall moderate level suggests that although patients demonstrate awareness of health‐promoting behaviors, there remains considerable room for improvement, particularly in behavior maintenance and sustainability. Clinically, suboptimal lifestyle engagement—especially in physical activity and diet‐related behaviors—may increase vulnerability to seizure triggers and compromise long‐term self‐management. Similarly, Szałwińska et al. (2021), Dayapoğlu et al. (2021), and Ayanda and Sulyman (2020) emphasized the importance of regular health checks and medication adherence for seizure control. However, studies by Du Rausas et al. (2024) and Strzelczyk et al. (2023) reported lower healthy lifestyle scores among epilepsy patients, highlighting the need for interventions to promote healthier behaviors. Notably, our moderate HPLP‐II total score contrasts with reports of lower scores in some studies. This discrepancy may be related to differences in sample characteristics (e.g., follow‐up setting, disease duration, sociodemographic profile), measurement periods, and contextual factors such as access to healthcare services and cultural norms influencing self‐report. These variations highlight that lifestyle behaviors in epilepsy are context‐sensitive and should be interpreted within the population studied. Sauls et al. (2024) and Robinson et al. (2008) noted that improving health responsibility can enhance the quality of life for these patients. These findings suggest that holistic approaches, including educational programs and support groups, are crucial in managing epilepsy. However, beyond general education, structured and measurable nurse‐led intervention components should be clearly defined. These may include individualized physical activity prescriptions adapted to seizure risk, diet adherence monitoring plans, medication adherence counseling, seizure diary tracking, and scheduled follow‐up assessments. From a broader perspective, these findings reflect the complex interaction between demographic characteristics, perceived health status, and behavioral self‐regulation in chronic neurological conditions.

The study found that patients aged 26 and over, women, married individuals, those living in urban areas, housewives, those who follow their diet, and those with better health responsibility had higher scores (Table 3). Older individuals are more conscious about health issues and prioritize regular check‐ups. Szałwińska et al. (2021) reported that age significantly affects health responsibility, with older patients attending health checks more regularly. Similarly, Rathinam et al. (2024) highlighted that older individuals have higher awareness of nutrition and health responsibility. Women are generally more sensitive to health issues, apply for health services more frequently, and prioritize regular health checks (Sauls et al. 2024). However, Tombini et al. (2021) found that health responsibility was lower in women. Married individuals benefit from spousal support, which helps increase their health responsibility and encourages regular attendance at health checks. Du Rausas et al. (2024) emphasized that married individuals' stronger social support networks enhance health responsibility. These findings suggest that demographic factors play a crucial role in health responsibility and should be taken into account in intervention planning.

Individuals living in urban areas have higher health responsibilities due to easier access to health services, as noted by Rathinam et al. (2024) and Szałwińska et al. (2021). Urban residents benefit from more extensive health service opportunities compared to rural populations, which supports this finding. Housewives were found to have higher health responsibilities, likely due to the time they spend at home and their ability to focus on their health (Sauls et al. 2024). Additionally, housewives may have more time and flexibility to focus on their health and attend routine check‐ups. These factors collectively explain the higher health responsibility observed in housewives.

Individuals who follow their diet have higher general health responsibilities, attend regular health checks, and manage their health better (Sarlo et al. 2023). Szałwińska et al. (2021) emphasized the importance of dietary compliance in seizure control and improving general health status, highlighting it as an indicator of health awareness. Nutritional awareness is associated with greater health responsibility, as reported by Du Rausas et al. (2024), who noted that healthy dietary habits positively impact overall health and encourage regular health checks (Arıkan and Turan 2024). These findings underscore the pivotal role of nutritional habits in promoting health responsibility and overall well‐being.

This study shows that physical activity levels among patients with epilepsy are relatively low compared with other lifestyle domains (Table 2). This finding is particularly concerning, as reduced physical activity may contribute not only to poorer seizure control but also to long‐term cardiometabolic risks and diminished psychosocial functioning. From a neurobiological perspective, physical inactivity may increase systemic inflammation, impair mitochondrial function, and negatively affect neuroendocrine regulation, all of which may contribute to increased cortical excitability. Conversely, structured physical activity may enhance inhibitory neurotransmission and improve stress resilience, thereby potentially reducing seizure susceptibility and improving cortical excitability thresholds (Pimentel et al. 2015; Talo and Turan 2023; Collard and Ellis‐Hill 2017). This association may also be explained by Social Cognitive Theory, which suggests that health behaviors are influenced by the interaction of personal beliefs, environmental factors, and behavioral experiences. In epilepsy, fear of seizures, limited exercise‐related knowledge, and low self‐efficacy may reduce physical activity participation. Nurse‐led education and support may improve self‐efficacy and outcome expectations, thereby encouraging safer and more sustainable engagement in physical activity. Concrete interventions should include risk‐assessed exercise planning, supervised initiation programs, written safety guidance, and gradual activity progression protocols to reduce fear‐related avoidance. Similarly, Talo and Turan 2023 reported low levels of physical activity among epilepsy patients. However, progressive muscle relaxation exercises were shown to improve their quality of life, making them a recommended complementary nursing intervention. Szałwińska et al. (2021) and Ayanda and Sulyman (2020) also reported lower physical activity levels in epilepsy patients compared to healthy individuals. Sauls et al. (2024) and Robinson et al. (2008) emphasized the importance of regular physical activity for overall health and seizure control, while Du Rausas et al. (2024) noted that low physical activity levels negatively impact the quality of life in patients with epilepsy. Additionally, higher physical activity levels were observed in single individuals, those with a university‐level education, civil servants, individuals who engaged in sports, and those who followed a healthy diet (Table 3). The greater activity levels among single individuals may be linked to their higher participation in social and support group activities, as highlighted by Rathinam et al. 2024.

Individuals with higher levels of education tend to have greater health awareness and a better understanding of the importance of physical activity (Pimentel et al. 2015). Szałwińska et al. (2021) and Du Rausas et al. (2024) reported that increased education levels enhance health consciousness and encourage participation in regular physical activities. The structured lifestyle of civil servants, combined with the motivation they receive from colleagues, may also contribute to higher physical activity levels. Sauls et al. (2024) emphasized that regular sports practice has a positive impact on the physical and psychological health of epilepsy patients, while also potentially reducing the frequency and severity of seizures (Yogarajah and Mula 2019). Additionally, individuals who adhere to a healthy diet tend to have higher health responsibilities and prioritize regular physical activity. Szałwińska et al. (2021) noted that dietary compliance improves general health and increases physical activity levels. These findings underscore the crucial role of education, professional structure, and healthy habits in fostering physical activity.

This study found that the nutritional habits of patients with epilepsy were moderate (Table 2). This moderate level suggests that patients may demonstrate some positive dietary behaviors, but these behaviors are not sufficiently regular, comprehensive, or sustained to be considered optimal. In the context of epilepsy management, this finding may indicate that nutritional practices are only partially integrated into daily self‐management and may therefore be insufficient to fully support seizure control, metabolic balance, and overall well‐being. Szałwińska et al. (2021) and Ayanda and Sulyman (2020) emphasized the need to improve dietary habits in patients with epilepsy and highlighted the effectiveness of a ketogenic diet in seizure control. Similarly, Sauls et al. (2024) and Robinson et al. (2008) noted that dietary improvements contribute positively to seizure control and overall health. Additionally, better eating habits were observed in patients who were aged 26 or older, married, primary school graduates, housewives, used three or more medications for epilepsy, followed their diet, described their sleep and nutrition patterns accurately, weighed between 61–75 kg, and had a normal BMI (Table 3). These findings underscore the importance of tailored dietary interventions in the management of epilepsy. Mechanistically, ketogenic and low‐glycemic dietary approaches may alter neuronal energy metabolism by promoting ketone body utilization, which has been associated with stabilization of neuronal membranes and reduced glutamatergic excitability (Liu et al. 2018; D'Andrea Meira et al. 2019). Adequate intake of antioxidant micronutrients may further reduce oxidative stress, which is known to contribute to epileptogenesis. Therefore, dietary quality may influence seizure dynamics through metabolic and neurochemical pathways. These metabolic effects may partly explain why diet adherence and perceived diet status emerged as significant predictors in the regression model.

Rathinam et al. (2024) reported that older individuals tend to have a greater awareness of nutrition and health responsibility, adopting healthier eating habits as they age. Similarly, Du Rausas et al. (2024) noted that married individuals benefit from stronger social support networks, which have a positive impact on their eating habits. Spouses often encourage more regular and healthy eating practices. While individuals with lower education levels were found to have more traditional eating habits, housewives generally have more nutritious and more regular eating patterns due to their active role in food preparation, as supported by Rathinam et al. (2024) and Du Rausas et al. (2024). Epilepsy patients with polypharmacy tend to pay closer attention to their diets to minimize drug side effects, as noted by Du Rausas et al. (2024) and Dayapoğlu et al. (2021). Dietary adherence also enhances seizure control and general health, as highlighted by Szałwińska et al. 2021. In addition, regular and appropriate antiseizure medication adherence is a critical determinant of seizure recurrence and quality of life. Therefore, intervention models should integrate medication adherence education, reminder systems, and periodic therapy review in collaboration with neurology teams. Individuals with healthy sleep patterns tend to exhibit better eating habits, as reported by Du Rausas et al. (2024), who found a positive relationship between sleep quality and nutrition. Sauls et al. (2024) further emphasized that individuals with a normal BMI have more regular and healthy eating habits. In terms of interventions, Talo and Turan 2023 found that progressive muscle relaxation exercises improved sleep quality in patients with epilepsy. Meanwhile, Arıkan and Turan (2024) demonstrated that Reiki practice enhanced sleep quality and overall quality of life in this population. These findings suggest that complementary nursing interventions such as relaxation exercises and Reiki can support both sleep and dietary habits in epilepsy management.

This study found that the Spiritual Growth level of epilepsy patients was at a relatively high level (Table 2). Szałwińska et al. (2021), Özcan and Çiftçi (2024), and Ayanda and Sulyman (2020) emphasized that spiritual support can improve the quality of life for epilepsy patients, while Sauls et al. (2024) noted its role in helping patients cope with stress. Additionally, those with good income levels, better perceived health, healthy sleep patterns, and balanced nutrition demonstrated higher Spiritual Growth (Table 3). Tsigebrhan et al. (2021) highlighted the significant impact of income level on Spiritual Growth and quality of life, as higher income allows individuals to devote more attention to health and spirituality. Ayanda and Sulyman (2020) stated that better self‐perceived health is associated with higher Spiritual Growth, which contributes to improved quality of life. Du Rausas et al. (2024) reported that healthy sleep has a positive impact on overall and general health. Similarly, Szałwińska et al. (2021) emphasized that a balanced diet supports Spiritual Growth. These findings suggest that both personal health behaviors and socioeconomic factors influence Spiritual Growth in individuals with epilepsy.

This study found that epilepsy patients had good Interpersonal Relations skills (Table 2). Szałwińska et al. (2021) and Ayanda and Sulyman (2020) highlighted that strong social support networks improve the quality of life of epilepsy patients, while Sauls et al. (2024) noted their contribution to seizure management and general health. Interpersonal Relations was better among patients aged 26 and over, women, married individuals, those with higher income, and those who perceived their health positively (Table 3). Rathinam et al. (2024) reported that older individuals benefit from wider social support networks, positively influencing Interpersonal Relations. Similarly, Du Rausas et al. (2024) noted that women generally have stronger social support networks, enhancing their communication skills. Marriage strengthens ties with spouses and family members, further expanding social support, as emphasized by Ayanda and Sulyman (2020). High‐income levels also enable greater participation in social activities, broadening social networks, as observed by Du Rausas et al. (2024) and Al Mheid and Quyyumi (2017). Lastly, Szałwińska et al. (2021) reported that epilepsy patients with better‐perceived health are more active socially, which contributes to stronger social support networks and improved health outcomes. These findings highlight the role of social support and demographic factors in enhancing Interpersonal Relations among individuals with epilepsy.

This study found that epilepsy patients were moderately successful in stress management (Table 2). Szałwińska et al. (2021) and Ayanda and Sulyman (2020) emphasized that stress is a critical trigger for epilepsy seizures, and teaching stress management techniques is essential. Sauls et al. (2024) and Robinson et al. (2008) noted that effective stress management can improve seizure control and quality of life in patients with epilepsy. Better stress management was observed among patients aged 26 and older, those with a good perceived health status, good sleep patterns, and a balanced diet (Table 3). Arıkan and Turan (2024) also reported similar findings. Du Rausas et al. (2024) stated that stress management skills improve with age, reducing seizure frequency, a finding supported by the literature, which links age to greater stress‐coping experience. Ayanda and Sulyman (2020) highlighted that healthier individuals can focus more on stress management techniques, which aid in controlling epilepsy seizures. Rathinam et al. (2024) reported that regular sleep patterns significantly improve stress management, reducing stress levels, while Szałwińska et al. (2021) emphasized the positive effects of a balanced diet on stress management. Healthy sleep and nutrition habits enhance an individual's ability to cope with stress, underscoring their importance in epilepsy management. The regression model explained 36% of the variance in healthy lifestyle behaviors (*R* ^2^ = 0.36), indicating that perceived health and diet‐related variables play a substantial role in shaping lifestyle engagement among adults with epilepsy. This indicates a moderate explanatory power, suggesting that additional psychosocial, environmental, or disease‐specific factors not included in the present model may also contribute to lifestyle behaviors. This finding supports the need for targeted intervention strategies focusing specifically on perceived health status and diet‐related behaviors, rather than broad non‐specific lifestyle recommendations. Integrating qualitative and quantitative approaches in future research may help minimize reporting bias and provide a more comprehensive understanding of lifestyle behaviors in epilepsy. The present findings further indicate that lifestyle dimensions in epilepsy are interconnected rather than independent. Improvements in one domain may create positive effects in others; for instance, better nutritional habits may contribute to improved stress regulation and sleep quality, whereas effective stress management may support dietary adherence and greater participation in physical activity. Likewise, physical activity may enhance emotional well‐being and sleep, thereby indirectly strengthening other self‐management behaviors. This multidirectional relationship highlights the importance of integrated nursing interventions that simultaneously target multiple lifestyle domains in order to achieve more sustainable behavioral change. The findings of this study have important implications for both nursing practice and health policy. By identifying physical activity, nutrition, perceived health, and diet adherence as critical lifestyle‐related areas, the study provides an evidence base for the development of more effective epilepsy management programs. In nursing practice, these findings support the design of structured, nurse‐led interventions focused on individualized lifestyle assessment, counseling, adherence monitoring, and behavioral support. In terms of health policy, the results suggest that epilepsy care should move beyond seizure‐focused management and incorporate routine lifestyle counseling, multidisciplinary follow‐up, and community‐based self‐management support programs. Such program models may contribute to improved seizure control, better quality of life, and more sustainable long‐term care for adults with epilepsy.

### 4.1 Study Limitations

This study has several limitations. First, the absence of a healthy control group limits the comparability of the results. This makes it challenging to contrast the findings with those of healthy individuals and may reduce the generalizability of the outcomes. Future studies are recommended to include healthy control groups to enable more comprehensive analyses. Second, the data in this study were based on participants' self‐reports, which may introduce biases such as social desirability bias or recall inaccuracies. Although data were collected through face‐to‐face standardized interviews in a private setting, self‐report measures inherently carry a risk of response bias. Future studies should incorporate triangulation methods, such as in‐depth interviews, observational approaches, or objective clinical indicators, to enhance measurement accuracy and strengthen the robustness of findings. Lastly, the research was conducted in a specific geographical region (Eastern Anatolia) and within a single university hospital. This single‐center design may also have contributed to center‐specific effects. In addition, the accessibility of healthcare services and the region's sociocultural characteristics may have influenced lifestyle behaviors, potentially limiting the transferability of the findings to other contexts. Therefore, the external validity of the findings may be limited, and the results should be interpreted with caution when applied to epilepsy populations in different regions or healthcare settings. Future multi‐center studies with larger and more heterogeneous samples across different geographic regions and healthcare settings are recommended to strengthen external validity and improve the generalizability of the findings. Including primary, secondary, and tertiary healthcare centers from diverse socioeconomic regions would further enhance the representativeness of future findings.

### 4.2 Clinical Relevance

This study indicates that adults with epilepsy show only moderate healthy lifestyle behaviors, highlighting the need for improvement. Nurses are well positioned to enhance self‐management by providing individualized lifestyle counseling, developing and regularly reviewing care plans, and promoting adherence to regular health check‐ups. Structured nurse‐led assessment tools based on HPLP‐II domains may facilitate early identification of vulnerable lifestyle areas, particularly physical activity. These tools may be operationalized through structured intervention protocols including: (1) medication adherence checklists, (2) seizure‐trigger monitoring forms, (3) individualized nutrition plans, (4) graded physical activity programs, and (5) stress‐management skill training modules. Nurse‐led interventions that strengthen social support, deliver stress management training, and incorporate spiritual support when appropriate may improve seizure management and quality of life. Integrating these strategies into nursing education and multidisciplinary practice can help address patients’ physical, psychological, and social needs more comprehensively.

### 4.3 Conclusion and Recommendations

In this study, it was found that epilepsy patients generally adhered to healthy lifestyle behaviors at a moderate level. Demographic factors were found to influence healthy lifestyle behaviors. It was determined that women, married people, those with higher levels of education, and those who attended regular health check‐ups had higher healthy lifestyle behavior scores. Similar trends were observed in subscales, including health responsibility, physical activity, nutrition, Spiritual Growth, Interpersonal Relations, and stress management.

Beyond confirming existing literature, these findings underscore the need to shift from general lifestyle advice to structured, behavior‐specific intervention models tailored to demographic and perceived health characteristics. In line with these findings, comprehensive education programs and support groups should be established to promote healthy lifestyle behaviors among individuals with epilepsy. Health professionals should provide counseling and guidance for the individual needs of patients. Additionally, strengthening social support networks and increasing access to health services may improve patients' quality of life. Diet and exercise programs should be regularly reviewed and updated to support seizure control and management. Future research should test structured, multi‐component intervention models in longitudinal or randomised controlled trials to determine their direct impact on seizure frequency and quality‐of‐life outcomes. Future intervention studies should explicitly incorporate structured medication management algorithms and adherence‐monitoring tools to assess their direct impact on seizure recurrence. Consideration of activities to support the Spiritual Growth of individuals with epilepsy may also have positive effects on stress management and overall well‐being.

## Author Contributions

B.Ç. and N.B. conceived and designed the study, collected the data, analyzed and interpreted the data, drafted and critically revised the manuscript, and read and approved the final manuscript.

## Funding

This research was supported by the Atatürk University Scientific Research Project (Project No: TYL‐2021‐969250WB2027).

## Ethics Statement

This study was conducted in accordance with the Declaration of Helsinki. Ethical approval was obtained from the Atatürk University Faculty of Medicine Ethics Committee (Approval No: B.30.2.ATA.0.01.00/169; Date: 15.04.2021). Written informed consent was obtained from all participants prior to data collection.

## Consent

The authors have nothing to report.

## Conflicts of Interest

The authors declare that they have no conflicts of interest.

## Data Availability

The datasets generated and/or analyzed during the current study are available from the corresponding author on reasonable request.
